# Reduction of Beta Cyclodextrin by Curd Washing in Low-Cholesterol Manchego Cheese

**DOI:** 10.3390/molecules28124709

**Published:** 2023-06-12

**Authors:** Leocadio Alonso, María V. Calvo, Javier Fontecha

**Affiliations:** 1 Instituto de Productos Lácteos de Asturias (CSIC), 33300 Villaviciosa, Asturias, Spain; 2 Instituto de Investigación en Ciencias de la Alimentación (CSIC-UAM), 28049 Madrid, Spain

**Keywords:** beta cyclodextrin, ewe’s milk, curd washing, cheese, Manchego, lipids, cholesterol

## Abstract

Beta-cyclodextrin (β-CD) is a cyclic oligosaccharide consisting of seven glucose units. β-CD is increasingly used in food research to reduce cholesterol due to its affinity for non-polar molecules such as cholesterol and as a natural additive. The purpose of this study was to evaluate the effect of curd washing in ewe’s milk cheese on the reduction in cholesterol by β-CD from pasteurized ewe’s milk Manchego cheese and the characteristics of its main components: milk, lipids, and flavor. An approximately 98.45% cholesterol reduction was observed in washed experimental cheeses that were treated by using β-CD. The remaining residual β-CD from the effect of curd washing was 0.15% in mature cheese, of the initial 1% β-CD treatment of the milk. The chemical properties (fat, moisture, and protein) did not change as a result of the curd washing with or without β-CD. The curd washing with or without β-CD on the levels of the various lipid fraction (fatty acids, triglycerides, and phospholipids) were comparable in treated and untreated cheeses. The effects of curd washing and the β-CD treatment did not significantly affect flavor components or short chain free fatty acids. The β-CD molecules were edible and nontoxic; as a result, they could be used safely in cholesterol removal processing in cheese manufacturing, improving the reduction in residual β-CD by curd washing by 85%. Therefore, the present study suggests that curd washing combined with β-CD is an effective process for cholesterol removal in Manchego cheese, preserving its desirable properties.

## 1. Introduction

Although dairy products in general have the image of being healthy foods [1], this image is often not perceived for products with a high fat content such as cream, butter, and cheeses due to the high amount of cholesterol and saturated fatty acids, mainly in mature cheeses with high fat content, and consequently, the reduction of cholesterol in these products and especially in matured cheeses would reduce the risk of cardiovascular diseases.

To reduce the risk of coronary heart disease, the World Health Organization and the American Heart Association advise consumers to consume fewer saturated fatty acids and cholesterol. A market for low cholesterol products has been generated by this advise and radical view points [2]. Dairy products with lower cholesterol is becoming more popular today. The removal of cholesterol from food can be accomplished using a variety of techniques, such as the incorporation of vegetable oils [3,4], distillation and crystallization [5,6], adsorption with saponin and digitonin [7,8], assimilation of cholesterol by microorganism enzymes [9,10], and removal by supercritical carbon

Dioxide extraction [11,12]. Several investigations have been detailing the usage of β-CD in food have been reported [13,14,15]. It has been demonstrated that β-CD molecules can be utilized to eliminate cholesterol from milk and dairy products in an efficient manner while being non-toxic and indigestible [15]. Seven glucose units make up the cyclic oligosaccharide known as β-CD, which is produced from starch by the enzyme cyclodextrin glycotransferase, which also breaks the polysaccharides chain to generate cyclic polysaccharide molecules.

The doughnut-shaped molecule of β-CD has a circular hydrophobic region in the center that is about the same size as a molecule of cholesterol. This gives the molecule an affinity for non-polar molecules like cholesterol. The cavity’s radius nearly perfectly fits a allowing the molecule to have a preference for non-polar compounds like cholesterol The cavity’s radius almost perfectly fits a cholesterol molecule, giving β-CD a high specificity for forming a 2:1 host/guest inclusion insoluble complex with cholesterol that can be separated by centrifugation or sedimentation [16,17].

One of Spain’s most recognizable hard cheeses is Manchego. It is produced by a designation of origin in the Castilla la Mancha region of Spain using only pure sheep’s milk [18]. Rich in fat (fat content in dry cheese is greater than 50%), Manchego has a distinctively strong flavor that develops over time. Although studies have been conducted to show that β-CD is a great ingredient for removing cholesterol from Manchego cheese [18], the potential effect of curd washing on this process has not yet been investigated. In the literature, we found works in which curd washing was used to control the lactose ratio in cheese making [19] or the insoluble Ca content [20]. However, little information is available on the effect of such curd washing on the chemical and organoleptic properties of cheese [21,22], and there are no data available in the particular case of β-CD-treated milk used in the manufacture of low-cholesterol cheeses.

The present study, which was a continuation of a previous research by Alonso et al. [18], aimed to assess the effect of the technological process of curd washing on the final quality of β-CD-treated Manchego cheese as well as on the reduction of residual β-CD throughout the manufacturing process of low-cholesterol Manchego cheese.

## 2. Results and Discussion

### 2.1. Gross Composition

In Table 1, the chemical composition and percentage of cholesterol removed from washed control cheese (WCC) and washed experimental cheese (WEC) containing 1% β-CD in milk are shown. Fat, moisture, and protein contents were not significantly different (*p* ≤ 0.05) between (WCC vs. WEC) respectively. Soluble nitrogen (SN) and non-protein nitrogen (NPN) were not significantly different (*p* ≤ 0.05) between WCC vs. WEC) (5.79 vs. 6.01% as protein) and (3.95 vs. 4.22% as protein); the slight increase in the proteolysis in WEC cheeses that may reflect a peptidase activity due to the influence of the β-CD but not significant [23,24]. The cholesterol removal rate of (WCC vs. WEC) (191.71 mg/100 g fat vs. 1.12 mg/100 g fat) reached a reduction of 98.45% in WEC. Similar cholesterol removal was also found by Kwak [25] in a study on the elimination of cholesterol using β-CD from Cheddar cheese and Kin [26] in Blue cheese. 0.15% of the original β-CD was left. It demonstrates that the gross composition of Manchego cheese made from ewe’s milk is unaffected by the elimination of cholesterol by β-CD or curd washing.

### 2.2. Lipid Characteristics

The mean percentages of fatty acids (%) for the washed control cheese (WCC) and the washed experimental cheese (WEC) containing 1% of β-CD in milk are shown in Table 2. When comparing the fatty acids from WCC vs. WEC cheeses, the amounts of fatty acids did not show any significant variations (*p* > 0.05). There have been investigations on the lipidic fraction’s impact on the production of low-cholesterol cheeses by β-CD. Chen et al. [27], when milk fat was fractionated to remove cholesterol using supercritical fluid extraction with carbon dioxide, it was found that the composition of the fatty acids in the fractionated milk fat differed significantly from that of the control cheeses. These authors reported that the extracted milk fat had 40% and 10% fewer short- and medium-chain fatty acids, respectively, than the control milk fat. Gonzalez-Hierro et al. [11] found similar results on the solubility of fatty acids in cream from ewe’s milk using supercritical fluid carbon dioxide. In our investigation, there was no statistically significant difference (*p* ≤ 0.05) between WCC and WEC for the proportion of short-chain (C4 to C8), medium-chain (C10 to C12) (8.34 vs. 8.26%), or long-chain (C14 to C18) fatty acids (82.90 vs. 82.44%) fatty acids. Alonso et al., 2019 [15] discovered comparable outcomes in a trial that used β-CD to lower the cholesterol content of milk fat.

The average triglyceride concentrations for each group of the washed control cheese (WCC) and the washed experimental cheese (WEC) containing 1% β-CD in milk are shown in Table 3. From C26 through C54, the triglycerides in the cheese fat were divided into 15 groups. The total of the various molecular triglyceride species with the same amount of carbon atoms made included each category. There were no differences between the two groups (WCC vs. WEC) (*p* ≤ 0.05). This was observed for the short-chain (C24-C32) triglycerides (8.18 vs. 8.01%), medium-chain (C34-C48) (76.98 vs. 77.99%), and long-chain (C50-C54) triglycerides (13.28 vs. 13.39%). Triglycerides in cheeses treated with β-CD to remove cholesterol have not been studied before. When fractionating milk fat to eliminate cholesterol using supercritical fluid extraction with carbon dioxide, Chen [27] noticed that the composition of the fatty acids in the fractionated milk fat differed significantly from that of the control cheeses. Because the triglycerides were removed by solvent extraction, which may have preferentially extracted some triglycerides better than others, the supercritical fluid extraction procedures used by these researchers may have produced some variation in the triglyceride composition.

Table 4 shows the phospholipid compositions of the washed control cheese (WCC), which does not contain β-CD, and the washed experimental cheese (WEC), which has 1% β-CD in milk. The relative composition of the various phospholipid classes in relation to the total phospholipids (PL) in the groups of cheeses (WCC vs. WEC) did not differ significantly (*p* ≤ 0.05) according to the analysis of variance. The most abundant phospholipid was phosphatidyethanolamine (45.72% of total PL vs. 40.61% of total PL), which was followed by phosphatidylcoline (32.04% of total PL vs. 29.20% of total PL) and sphyngomyelin (27.84% as total PL vs. 25.62% as total PL). Similar results were obtained by Alonso [15,18], in a study of the effect of β-CD on the phospholipids of the milk fat in pasteurized milk. More than 80% of the total phospholipids in dairy products came from these three species. The fact that β-CD particularly formed an inclusion complex with cholesterol [15] could be one of the explanations for why it did not influence various components of milk fat.

### 2.3. Flavor Characteristics

The flavor components extracted from the washed experimental cheese (WEC) with 1% of β-CD in milk and the washed control cheese (WCC) without β-CD are shown in Table 5. Both cheeses had a total of 13 flavor components, but there were notable variations between the samples. 13 flavoring compounds including five ketones, three aldehydes, and six alcohols, were found in all cheeses. The total amounts of ketones (2306.05 vs. 2141.87 mg/kg of cheese), aldehydes (1377.45 vs. 1472.90 mg/kg of cheese), and alcohols (4516.80 vs. 4590.10 mg·kg−1 of cheese) between (WCC vs. WEC) cheeses. Ethanol production was the highest among the flavor compounds measured, similar to those found by Kwak [25] in cheddar cheese treated with β-CD. In a study of cholesterol removed from cream cheese [28] by β-CD, found that the treated cheese’s flavor compounds did not differ from those of conventional cream cheeses in terms of total flavor compounds. Odd carbon number ketones exhibit normal odor features and low olfactory thresholds. These compound are created when fatty acids undergo beta-oxidation and decarboxylation. Aldehydes are known to be a minor component of cheeses due to their quick conversion to alcohols or the equivalent acids. The breakdown of branched-chain amino acids by an aminotransferase produces branched-chain aldehydes like 3-methyl butanal [29], which, together with ethyl, was the higher component in WCC and WEC.

Table 6 shows the quantities of short chain free fatty acids (SCFFAs), acetic, propionic, butyric, and caproic acids in washed control cheese (WCC) and washed experimental cheese (WEC) with 1% β-CD in milk. No difference was statistically significant (*p* ≤ 0.05) between the total SCFFAs (189.71 vs. 192.43 mg·kg^−1^ of cheese) and individual amounts of SCFFAs at the end of the three-month ripened cheeses. Similar results were found in the production in the amount of SCFFAs in Cheddar cheese produced by β-CD and the cholesterol reduction by [25,30]. Manchego cheese’s is strengthened by the release of butyric and caproic acid throughout the three-month ripening process.

The sensory qualities of the washed control cheese (WCC) and the washed experimental cheese (WEC) containing 1% β-CD in milk were assessed on a scale of 1 to 5. There were no variations (*p* ≤ 0.05) in flavor (2.97 vs. 2.86), aroma (2.88 vs. 2.80), color (3.49 vs. 3.37), or texture (3.29 vs. 3.03), and acceptability (3.22 vs. 3.18) between (WCC vs. WEC) cheeses. The texture was slightly different; this could be due to a slight increase in the moisture in the cheese treatment with β-CD due to the fact that the β-CD exterior was hydrophilic due to the ring with OH groups, where the interior cavity was relatively hydrophobic. As a result, β-CD can accommodate hydrophobics molecules inside the cavities as water molecules [31], which resulted in slow drainage but not significant. Over the period of the three-month ripening process, the overall preference remained constant, and there were no differences between (WCC vs. WEC) for any sensory qualities. According to this study, the majority of the sensory traits and overall preferences were comparable between the control group and the three-month-ripened cheese curd that had been washed and treated with β-CD. The cheeses treated with β-CD combined with the curd washing maintained the nutritional properties, in addition to a significant reduction in cholesterol and low remaining β-CD.

## 3. Materials and Methods

### 3.1. Chemicals

α-cyclodextrin (α-CD), β-cyclodextrin (β-CD), and all reagents were supplied by Sigma (St. Louis, MO, USA). Deionized water was prepared by a water purification system (Millipore Co., Burlington, MA, USA).

### 3.2. Manchego Manufacture

An amount of 100 L of pasteurized ewe’s milk from Monte de Toledo (Toledo, Castilla la Mancha, Spain) (15 s at 72 °C) containing 1% of β-CD (*wt*/*vol*) was placed in a cold room at 4 °C and mixed by stirrer for 30 min. After mixing, the treated milk was left static overnight at 4 °C for binding the cholesterol to the β-CD. A cholesterol-β-CD complex was formed and was sedimented by precipitation [15].

Manchego cheese was manufactured with pasteurized ewe’s milk (15 s at 72 °C) in two experiments, each one performed in triplicate. In each experiment, 100 L of pasteurized refrigerated whole milk was treated with/without BCD. Milk at 30 °C was inoculated at rate of 1% (*wt*/*wt*) of commercial mixed of *L. lactis* strains. After 20 min, 5 mL of rennet (Maxiren 1:15,000 strength *vol*/*vol*; Gist Brocades, Deft, The Netherland) was added to each vat of milk, renneting the milk in 40 min. The washed control cheese (WCC) without β-CD and the washed experimental cheese (WEC) with 1% of β-CD in the milk were made by washing the curd with deionized water twice. The gel was cut into 10 mm cubes, left in the curd/whey mixture for 15 min, and then stirred at 37 °C for 10 min. Stirring was then stopped, and the whey was removed and replaced by the same volume of deionized water at 37 °C. The curd–water mixture was again stirred for 15 min at 37 °C and drained off. The cheeses were pressed for 20 h at 20 °C into molds, brine salted at 12 °C for 24 h, and ripened for three months at 14 °C and 85–90% relative humidity [32].

### 3.3. Gross Composition

The fat, moisture, pH, and protein contents and nitrogen fractions were determined using the method in [33].

### 3.4. Beta Cyclodextrin Analysis

The Alonso 2008 method [34] was used to analyze CD. The internal standard for quantitative analysis, 5 mg of β-CD dissolved in 1 mL of water, was added to a sample of 10 g of cheese. It was shaken for two minutes at 40 °C, centrifuged for thirty minutes at ambient temperature at 40,000 rpm to separate the upper layer, and then the upper layer was filtered through a 0.45 μm Millipore Co. membrane. The internal standard-spiked supernatant was delivered to the autosampler in a 30 μL aliquot. 10 μL aliquot of the supernatant was injected for high performance liquid chromatography (HPLC) analysis.

The Waters Empower 2 chromatography data program (Waters, Milford, MA, USA) was used for data collecting and analysis during the HPLC analysis using a Waters Alliance 2695 separation module connected to a 410 refractive index (RI) detector. On a YMC ODS AQ column (Teknochroma, Barcelona, Spain), separation was done. Methanol and water (7:93) in an isocratic environment and at a flow rate of 1 mL/min made up the mobile phase composition. The internal standard was used to prepare the standard solutions in water to determine the elution time, and the sample peak area of β-CD was compared to the internal standard to determine the β-CD’s quantity.

### 3.5. Lipid Extraction

A process described by an International Standard Method for Milk and Milk Method was used to extract lipids from samples [35]. It involved adding an ammonia ethanol solution to a test portion and then extracting the lipids with diethyl ether and hexane. The solvent then entirely evaporated and the top layer was removed. The lipid extracts were collected, put into amber glass vials, flushed with nitrogen, and kept at 20 °C until analysis.

### 3.6. Determination of Cholesterol

The method used for cholesterol analysis was with capillary gas chromatography (GC) with direct injection of milk fat as described by Alonso 1995 [36]. About 30 mg of anhydrous milk fat were dissolved in 1 mL of hexane together with 0.1 mL of 5 cholestane as the internal standard (3.5 mg/mL in hexane) to be used in GC analysis. The Agilent Technology 6890 chromatograph (Palo Alto, CA, USA) was used for the GC analysis for free cholesterol using direct injection with flame ionization detector. An HP 5 fused silica capillary column (30 m × 0.32 mm i.d. 0.25 mm thickness) was employed for the analyses. 

### 3.7. Fatty Acids and Triglycerides Analysis

Fatty acid methyl esters (FAMES) were prepared by alkaline catalyzed methanolysis of the extracted lipids using 2 N KOH in methanol. An Agilent Technology 6890 chromatograph (Palo Alto, CA, USA) equipped with a FID detector, were used for analysis. The technique reported by Alonso et al., 1999 [37] was utilized to separate fatty acids using a CP Sil 88 fused silica capillary column (50 m × 0.25 mm i.d. 0.2 m film thickness, Chrompack, CA, USA). The technique described by Alonso et al., 1999 [37] was utilized to separate fatty acids using a CP-Sil 88 fused-silica capillary column (50 m × 0.25 mm i.d. 0.2 m film thickness, Chrompack, CA, USA). A flame ionization detector-equipped Agilent gas chromatograph 6890 (Palo Alto, CA, USA) was used for the GC analysis of triglycerides. The method described by Alonso 1993 [38] was utilized to undertake the analyses using a WCOT fused silica capillary column (25 m × 0.25 mm 0.1 m film thickness) coated with OV 17 TRI.

### 3.8. Phospholipid Analysis

Using 2 g of freeze-dried cheese sample, 2 g of sea sand, and loading a stainless steel extraction cell coated with filters on both sides, cheese fat was extracted using an Accelerated Solid Extraction ASE 200 extractor (Dionex Corp., Sunnyvale, CA, USA). Dichloromethane methanol solution (2:1, *vol*/*vol*) was used as the solvent mixture and 10.3 MPa of pressure was a fixed condition during the extraction to provide the highest cheese fat yield possible [39].

The separation of lipid classes was accomplished in an HPLC system (model 1260; Agilent Technologies Inc.) coupled with an evaporative light scattering detector (SEDEX 85 model; Sedere SAS, Alfortville Cedex, France) using the method [39].

### 3.9. Analysis of Volatile Compounds

The analysis of the volatile fraction was performed by headspace gas chromatography-mass spectrometry (GC-MS) described by Alonso et al., 1999 [40]. 

### 3.10. Short-Chain Free Fatty Acids

Cheese samples (1 g) were homogenized in 20 mL of distilled water, centrifuged at 10,000 rpm for 10 min, and filtered through a 0.40 μm filter to determine short chain free fatty acids (SCFFAs). Analyses were performed using a Hewlett Packard model 5890 A instrument with a flame ionization detector on a capillary silica column (HP FFAP, 30 m × 0.25 mm ID, 0.25 μm film thickness, Agilent J & W). Each peak area of a specific FFA was compared to the peak area of 2 ethyl butanoic acid as an internal standard for the quantitative analysis. 

### 3.11. Sensory Analysis

Twenty-two trained sensory panelists evaluated randomly coded cheeses. The flavor, aroma, color, texture, and acceptability were evaluated on a five-point scale (1 = poor, 5 = excellent).

### 3.12. Statistical Analysis

Analysis of variance (ANOVA) was applied to the experimental data using SAS statistical software (version 8.02, SAS Institute Inc., Cary, NC, USA). A Student’s t test was used for statistical analysis, and a (*p* ≤ 0.05) was used to indicate statistical significance.

## 4. Conclusions

The aim of this study was to evaluate the effect of curd washing on the reduction in cholesterol induced by β-CD in pasteurized ewe’s milk Manchego cheese, considering the main components of milk, lipids, and flavor characteristics in regular Manchego cheese. An approximately 98.45% cholesterol reduction was observed in the washed experimental cheeses that were treated with β-CD. The remaining residual β-CD from the effect of curd washing was 0.15%. The chemical properties (fat, moisture, and protein) did not change as a result of curd washing with or without β-CD. The amounts of different components of the lipid fraction (fatty acids, triglycerides, and phospholipids) were similar in both the treated and the untreated cheese as a result of curd washing and with or without β-CD. The β-CD had no effect on the majority of flavor components and short chain free fatty acids. When curd washed with or without CD, there were no differences in the sensory qualities (flavor, aroma, color, texture, and acceptability). The β-CD molecules are edible and non-toxic, they can be employed safely in the cheese manufacturing process to remove cholesterol, which results in an 85% reduction in residual β-CD after curd washing. Therefore, the results of the current investigation revealed that removing cholesterol from Manchego cheese while maintaining its chemical qualities may be accomplished by treating β-D with the effect of curd washing improving the reduction in residual β-CD.

## Figures and Tables

**Table 1:** Gross composition of the controls and the experimental Manchego cheeses by curd washing and β-CD treatment of milk.

Parameter	WCC	WEC
Fat (%)	32.51 ± 1.18 ^a^	31.53 ± 1.13 ^a^
Moisture (%)	37.15 ± 1.93 ^a^	36.46 ± 1.70 ^a^
Protein (%)	25.10 ± 1.16 ^a^	24.96 ± 1.05 ^a^
SN (% as protein)	5.79 ± 0.32 ^b^	6.01 ± 0.35 ^b^
NPN (% as protein)	3.95 ± 0.24 ^b^	4.22 ± 0.41 ^b^
pH	4.85 ± 0.25 ^a^	5.30 ± 0.21 ^a^
Cholesterol (mg/100 g fat)	191.71 ± 0.19 ^a^	1.12 ± 0.14 ^a^
Cholesterol removal (% fat)	-	98.45 ± 5.12 ^b^
Remaining β-CD (%)	-	0.15 ± 0.11 ^b^

WCC: washed control cheese; WEC (1% β-CD): washed experimental cheese. β-CD: Beta-cyclodextrin. SN: soluble nitrogen (% as protein); NPN: non-protein nitrogen (% as protein);. ^a,b^ Different letters in the same row mean significant differences (*p* ≤ 0.05). Mean standard deviation (*n* = 3).

**Table 2:** Fatty acid composition (g/100 g fat) of the control and experimental Manchego cheeses with the effects of curd washing and β-CD treatment of milk.

Fatty Acid	WCC	WEC
C4:0	2.18 ± 0.22 ^a^	2.16 ± 0.24 ^a^
C6:0	1.68 ± 0.08 ^a^	1.67 ± 0.05 ^a^
C8:0	1.66 ± 0.07 ^a^	1.64 ± 0.06 ^a^
C10:0	4.95 ± 0.18 ^a^	4.91 ± 0.16 ^a^
C10:1	0.25 ± 0.05 ^a^	0.24 ± 0.05 ^a^
C12:0	3.14 ± 0.16 ^a^	3.11 ± 0.14 ^a^
C14:0	9.21 ± 0.92 ^a^	9.14 ± 0.42 ^a^
C14:1	0.88 ± 0.08 ^a^	0.89 ± 0.04 ^a^
C15:0	0.25 ± 0.04 ^a^	0.23 ± 0.07 ^a^
C16:0	27.41 ± 1.13 ^a^	27.21 ± 1.11 ^a^
C16:1	0.77 ± 0.15 ^a^	0.71 ± 0.13 ^a^
C17:0	0.58 ± 0.09 ^a^	0.52 ± 0.05 ^a^
C18:0	13.59 ± 0.65 ^a^	13.56 ± 0.39 ^a^
C18:1t	2.65 ± 0.26 ^a^	2.78 ± 0.14 ^a^
C18.1c	22.93 ± 1.06 ^a^	22.78 ± 1.21 ^a^
C18:2	3.26 ± 0.31 ^a^	3.26 ± 0.18 ^a^
C18:3	0.40 ± 0.06 ^a^	0.39 ± 0.03 ^a^
C18.2 (c9t11)	0.97 ± 0.08 ^a^	0.97 ± 0.05 ^a^

WCC: washed control cheese; WEC (1% β-CD): washed experimental cheese. β-CD: Beta-cyclodextrin. ^a^ Different letters in the same row mean significant differences (*p* ≤ 0.05). Mean standard deviation (*n* = 3).

**Table 3:** Triglyceride composition (g/100 g fat) of control and experimental Manchego cheeses with the effects of the curd washing and β-CD.

Triglyceride	WCC	WEC
C24	0.32 ± 0.08 ^a^	0.31 ± 0.06 ^a^
C26	0.80 ± 0.05 ^a^	0.77 ± 0.08 ^a^
C28	1.44 ± 0.13 ^a^	1.42 ± 0.17 ^a^
C30	2.47 ± 0.21 ^a^	2.50 ± 0.29 ^a^
C32	3.15 ± 0.39 ^b^	3.01 ± 0.45 ^b^
C34	5.04 ± 0.48 ^a^	5.09 ± 0.56 ^a^
C36	7.04 ± 0.54 ^a^	7.18 ± 0.50 ^a^
C38	10.65 ± 1.30 ^a^	11.01 ± 1.39 ^a^
C40	17.89 ± 1.32 ^a^	17.15 ± 1.42 ^a^
C42	16.17 ± 1.50 ^a^	16.21 ± 1.68 ^a^
C44	7.44 ± 0.66 ^a^	8.89 ± 0.51 ^a^
C46	7.04 ± 0.52 ^a^	7.16 ± 0.59 ^a^
C48	5.71 ± 0.49 ^a^	5.30 ± 0.54 ^a^
C50	4.39 ± 0.51 ^a^	4.55 ± 0.62 ^a^
C52	4.31 ± 0.56 ^a^	4.24 ± 1.57 ^a^
C54	4.58 ± 0.45 ^a^	4.60 ± 0.41 ^a^

WCC: washed control cheese; WEC (1% β-CD): washed experimental cheese. β-CD: Beta-cyclodextrin. ^a,b^ Different letters in the same row mean significant differences (*p* ≤ 0.05). Mean standard deviation (*n* = 3).

**Table 4:** Phospholipid composition of the control and experimental Manchego cheeses with the effects of curd washing and β-CD treatment of milk.

Phospholipids	WCC	WEC
Total PLs (mg/100 g of fat)	0.05 ± 0.01 ^a^	0.09 ± 0.01 ^a^
PE (% of PL)	45.72 ± 5.12 ^a^	40.61 ± 1.53 ^a^
PI (% of PL)	2.95 ± 0.30 ^a^	2.53 ± 1.51 ^a^
PS (% of PL)	1.68 ± 0.35 ^a^	2.10 ± 1.31 ^a^
PC (% of PL)	32.04 ± 1.40 ^a^	29.20 ±1.80 ^a^
SM (% of PL)	27.84 ± 3.83 ^a^	25.62 ± 3.22 ^a^

WCC: washed control cheese; WEC (1% β-CD): washed experimental cheese. β-CD; Beta-cyclodextrin. PLs: phospholipids; PE: phosphatidylethanolamine; PI: phosphatidylinositol; PS: phosphatidylserina; PC: phosphatidylcoline; SM: sphyngomyelin. ^a^ Different letters in the same row mean significant differences (*p* ≤ 0.05). Mean standard deviation (*n* = 3).

**Table 5:** Volatile compounds (mg·kg^−1^ of cheese) of the controls and the experimental Manchego cheeses with the effects of curd washing and β-CD treatment of milk.

Compounds	WCC	WEC
Ketones		
2 Propanone	381.05 ± 26.89 ^a^	369.96 ± 25.32 ^a^
2 Butanone	20.16 ± 4.21 ^a^	21.45 ± 4.69 ^a^
2,3 Butanodione	1145.81 ± 56.38 ^a^	1041.65 ± 59.98 ^a^
2 Heptanone	512.18 ± 22.78 ^a^	496.21 ± 30.21 ^a^
3 Hydroxy 2 butanone	248.70 ± 20.09 ^a^	212.60 ± 24.60 ^a^
Aldehydes		
3 Methyl butanal	1358. 96 ± 70.32 ^b^	1452.95 ± 77.80 ^b^
Hexanal	13.54 ± 4.09 ^a^	15.74 ± 4.96 ^a^
Nonanal	4.95 ± 1.19 ^a^	4.21 ± 1.29 ^a^
Alcohols		
2 Propanol	10.64 ± 3.70 ^a^	9.65± 3.96 ^a^
Ethanol	4385.30 ± 95.79 ^b^	4456.02 ± 109.10 ^b^
2 Methyl 1 propanol	55.66 ± 7.80 ^a^	58.69 ± 7.02 ^a^
2 Butanol	25.69 ± 5.56 ^a^	23.20 ± 5.04 ^a^
2 Heptanol	39.57 ± 6.12 ^a^	42.54 ± 5.52 ^a^

WCC: washed control cheese; WEC (1% β-CD): washed experimental cheese. β-CD: Beta-cyclodextrin. ^a,b^ Different letters in the same row mean significant differences (*p* ≤ 0.05). Mean standard deviation (*n* = 3).

**Table 6:** Short-chain free fatty acids (SCFFAs) (mg·kg^−1^ of cheese) of the control and experimental Manchego cheeses with the effects of curd washing and β-CD treatment of milk.

SCFA	WCC	WEC
Acetic	133.06 ± 6.19 ^a^	129.53 ± 8.96 ^a^
Propionic	35.36 ± 4.96 ^a^	38.02 ± 4.90 ^a^
Butyric	17.32 ± 3.60 ^a^	21.16 ± 3.93 ^a^
Caproic	3.96 ± 3.12 ^a^	3.72 ± 3.61 ^a^

WCC: washed control cheese; WEC (1% β-CD): washed experimental cheese. β-CD: Beta-cyclodextrin. ^a^ Different letters in the same row mean significant differences (*p* ≤ 0.05). Mean standard deviation (*n* = 3).

## Data Availability

The data are available from the corresponding author.
